# The Hidden Variable: A Case of Dasatinib-Induced Respiratory Failure

**DOI:** 10.7759/cureus.11892

**Published:** 2020-12-04

**Authors:** Dimitrios Drekolias, Naga Vaishnavi Gadela, Asma Syeda, Jason Jacob

**Affiliations:** 1 Internal Medicine, University of Connecticut Health, Farmington, USA; 2 Internal Medicine, University of Connecticut, Farmington, USA; 3 Internal Medicine, Hartford Hospital, Hartford, USA

**Keywords:** acute lymphoblastic leukemia (all), philadelphia chromosome, dasatinib, respiratory failure

## Abstract

Tyrosine kinase inhibitors that target the BCR/ABL mutation have been used as therapies of BCR/ABL positive acute lymphoblastic leukemia (ALL) with significant results. Dasatinib is a multitargeted tyrosine kinase inhibitor with significant activity in Philadephia positive ALL which is resistant to imatinib, as well as in treatment-naïve patients. We present a case of an elderly patient with Philadelphia chromosome-positive ALL, who presented with acute hypoxic respiratory failure in the setting of active immunotherapy with dasatinib.

## Introduction

Acute lymphoblastic leukemia (ALL) is primarily a disease of the pediatric population affecting mainly ages younger than six years. There is a second epidemiological peak in adults older than 60 years [1,2]. The annual incidence of ALL is approximately 1-5/100,000. More than 60% of the cases are of the B-cell phenotype. It has been noted that B-ALL is occurring slightly more frequently in males compared to females. The Hispanic population appears to have the highest incidence among ethnic groups and the White population is affected approximately three times more than the African American population [3]. The BCR/ABL translocation is infrequent in ALL. The tyrosine kinase inhibitors targeting this mutation have been used in the treatment of BCR/ABL positive ALL cases. We present a case of an elderly patient with Philadelphia chromosome-positive ALL, who presented with acute hypoxic respiratory failure in the setting of active immunotherapy with the tyrosine kinase inhibitor, dasatinib.

## Case presentation

A 78-year-old male patient with a history of a recent diagnosis of acute lymphoblastic leukemia on immunotherapy, prostate cancer, lower extremity deep venous thrombosis with inferior vena cava filter, and hypertension, presented to the emergency department after developing progressively worsening dyspnea, cough, and fever of approximately one-week duration.

The patient had a recent admission approximately two months prior to this presentation. At that time, he endorsed loss of appetite and weight loss, accompanied by significant leukocytosis (white blood cell count of 72,900/uL) and thrombocytopenia (platelet count of 34,000/uL). Peripheral smear was suggestive of ALL. Flow cytometry revealed approximately 85-90% blasts, which expressed B-cell lineage markers (positive for HLA-DR, TdT, CD10, CD34, C19, CD20, CD22, and CD79a), consistent with the diagnosis of B-cell ALL. Cytogenetic analysis was also obtained and revealed the BCR/ABL translocation (the Philadelphia chromosome) as well as 9p21 homozygous deletion. He was subsequently started on the tyrosine kinase inhibitor, dasatinib.

**Figure 1:**
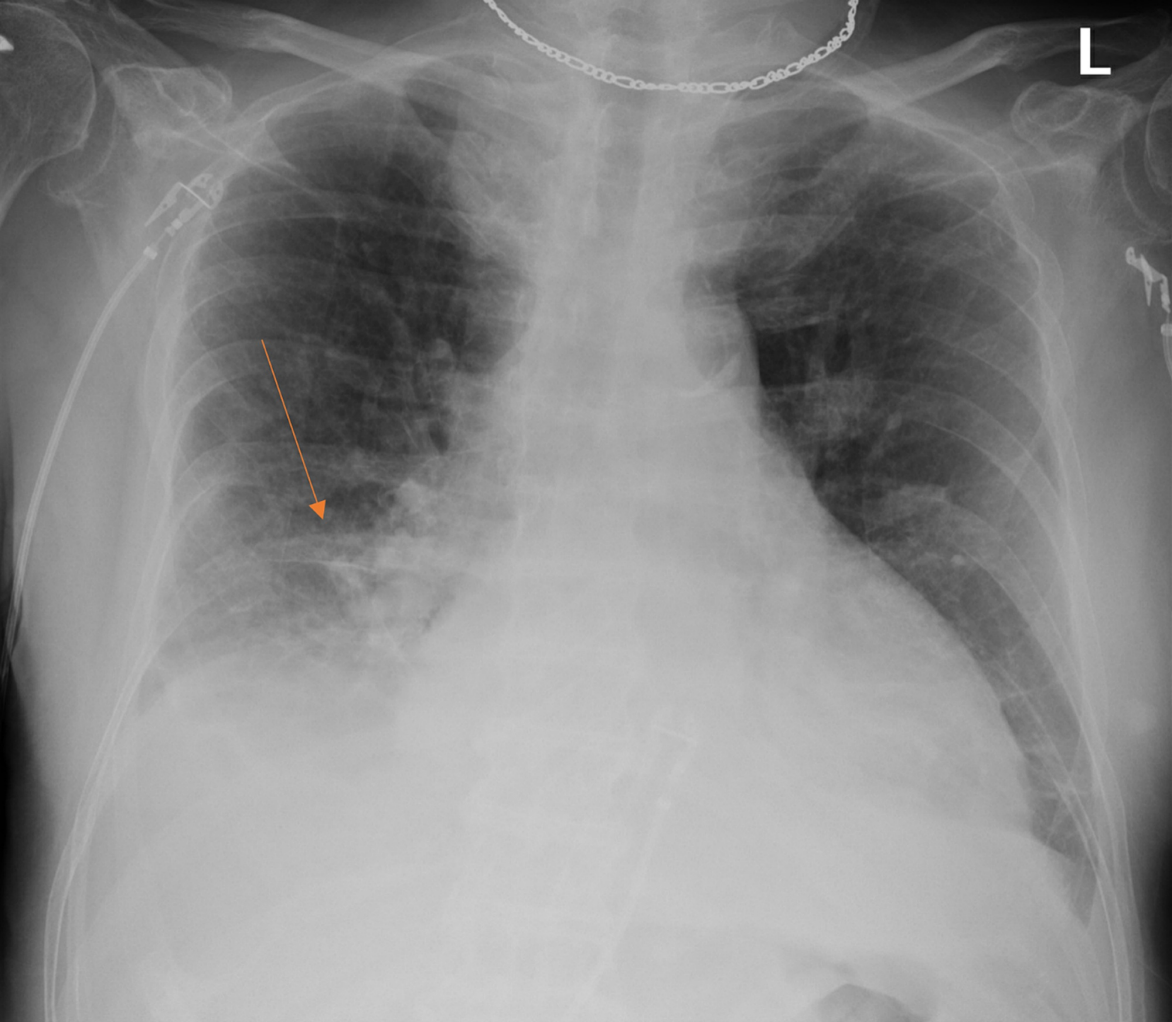
Chest x-ray, with haziness and streaky densities at the right lung base noted (orange arrow), concerning atelectasis. Left basilar linear densities also present, favoring atelectasis. However, no opacities were noted.

**Figure 2:**
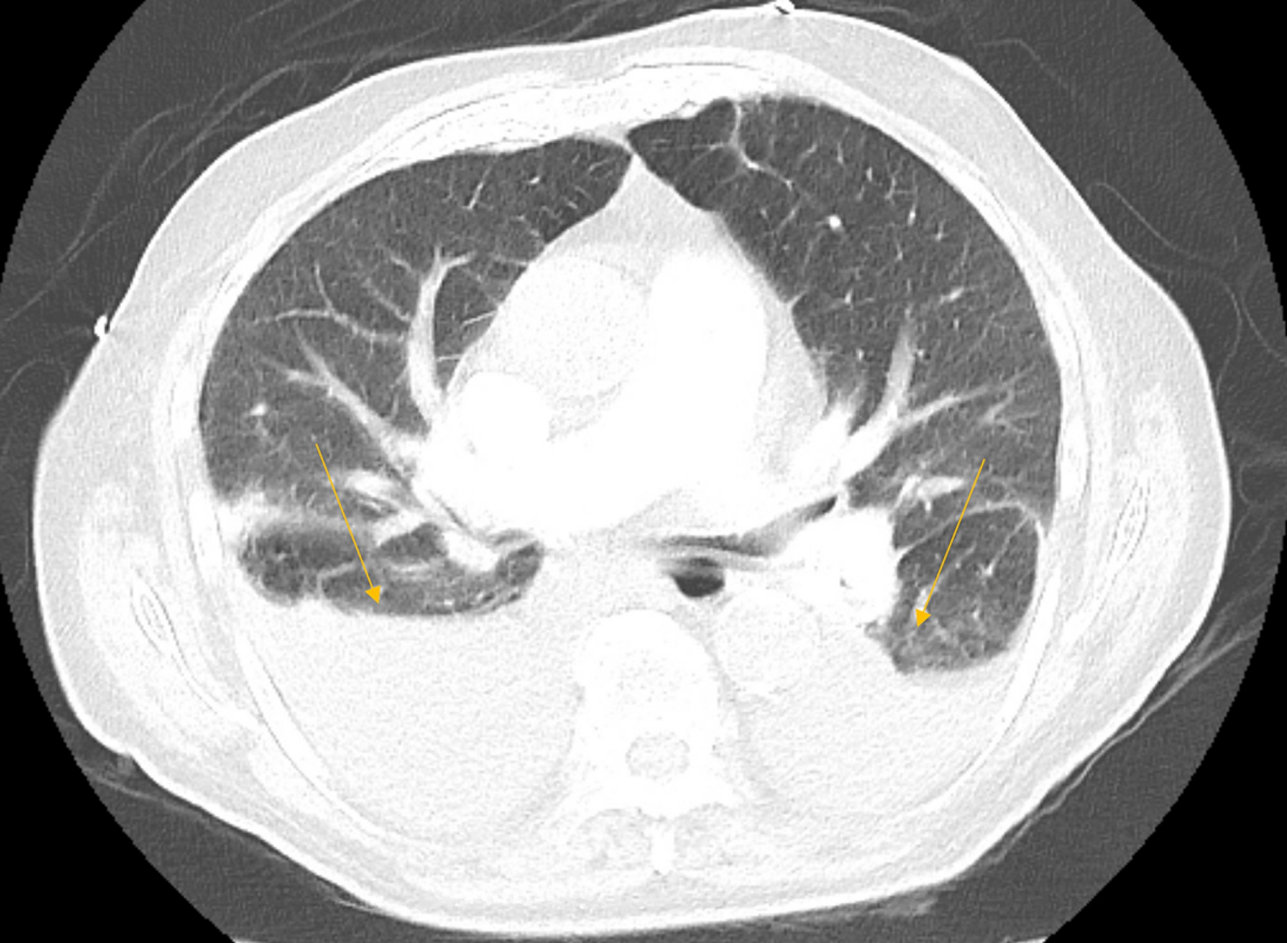
CT angiography revealed bilateral pleural effusions (orange arrows) with no signs of any inflammatory process and no pulmonary embolism.

During this admission, the patient denied any recent sick contacts and reported a recent COVID-19 negative test. In the emergency department, he was febrile to 102.9°F, tachycardic to 143, tachypneic with 22-34 breaths/min, maintaining oxygen saturation of 93% on 6L nasal cannula supplementation. The physical examination did not reveal any signs of volume overload with no crackles and no jugular venous distention or lower extremity edema present. Chest x-ray was obtained given concern for pneumonia, with haziness and streaky densities at the right lung base noted, concerning for atelectasis. Left basilar linear densities were also present, favoring atelectasis. However, no opacities were noted (Figure 1). CT angiography of the chest (CTA of the chest) was obtained for pulmonary embolism evaluation and revealed bilateral pleural effusions with no signs of any inflammatory process or pulmonary embolism (Figure 2). The patient was initially placed on broad-spectrum antibiotics with de-escalation to ceftriaxone and doxycycline. Bilateral thoracentesis was performed draining approximately 800cc. The pleural fluid analysis was consistent with transudate. Pleural fluid cytology was negative for malignant cells. The rest of the infectious workup was negative, including negative urinalysis and negative blood cultures. COVID-19 test was negative. Procalcitonin was 0.21ng/mL. Troponin was negative. Transthoracic echocardiogram was also obtained revealing biatrial dilation and a left ventricular ejection fraction of 69%. Given the absence of apparent etiology for the development of the pleural effusions, the decision was made to hold dasatinib. Subsequently, the patient was able to be weaned off oxygen supplementation to room air. He remained afebrile with no subsequent development of pleural effusions and was switched to nilotinib for management of his acute lymphoblastic leukemia.

## Discussion

The etiology of ALL is currently unknown. The existing hypotheses point toward pathogenesis related to ionizing radiation and/or currently unidentified infections [4]. Familial ALL, although a rare phenomenon, has been associated with inherited mutations of TP53, PAX5, and ETV6 [5,6]. Studies have also shown an increased incidence of B-ALL in patients with Down syndrome [7]. Certain single nucleotide polymorphisms (GATA3, ARID5B, IKZF1, CEBPE, CDKN2A/B) have also been associated with ALL [8,9]. The identification of the BCR/ABL fusion chromosome (Philadelphia chromosome) is also important in order to determine cases of BCR/ABL positive ALL [10]. The Philadelphia chromosome is detected and approximately 25% of adult ALL. It is very rare in pediatric cases, occurring in only 2-4% of cases. The incidence of the mutation increases with age, occurring in 20-30% of ALL patients older than 60 years old [11-13]. The Philadelphia chromosome in ALL used to be an adverse prognostic indicator, however, given the advent of tyrosine kinase inhibitor therapy, its prognostic contribution is currently unclear [14].

Flow cytometry is essential for the diagnosis of ALL. The immunophenotype needs to be defined by flow cytometry and/or immunohistochemistry. The material can be circulating lymphoblasts, bone marrow biopsy, or lymph nodes. Cytogenetic analysis consisting of conventional karyotype with chromosomal banding, with or without fluorescence in situ hybridization (FISH), along with molecular analysis for determination of gene expression patterns is part of the diagnostic evaluation of ALL. It should be performed on either the circulating lymphoblasts, the bone marrow biopsy specimen, or the lymph node for classification purposes.

Dasatinib is a multitargeted tyrosine kinase inhibitor with significant activity in Philadephia positive ALL resistant to imatinib. It has also shown significant result in treatment-naïve patients [15,16]. It has activity against many BCR/ABL kinase domain mutations that are resistant to imatinib and may be present at low levels at the time of Philadelphia positive ALL diagnosis [17,18]. In combination with chemotherapy, dasatinib results in complete remission rates of approximately 90 to 95 percent [15,19]. Common side effects include myelosuppression and platelet dysfunction. Pleural effusions, either unilateral or bilateral, have been reported with the use of dasatinib, with reported percentages being in the range of 15-30% of cases [20]. QT segment prolongation has been noted. Of note, contrary to imatinib, dasatinib penetrates the blood-brain barrier. In our case, the presence of bilateral pleural effusions in the absence of other apparent etiology should highlight the importance of keeping in mind dasatinib as a potential causative agent.

## Conclusions

In our case, the patient presented with a clinical picture concerning acute hypoxic respiratory failure secondary to infectious etiology. Extensive infectious workup was negative. Heart failure evaluation was also unrevealing. Our heightened awareness about the adverse effect profile of dasatinib assisted with the prompt and appropriate diagnosis of the cause of the patient's symptomatology and led to the resolution of the patient's symptoms upon discontinuation of the medication.
